# Knowing the unknown: The underestimation of monkeypox cases. Insights and implications from an integrative review of the literature

**DOI:** 10.3389/fmicb.2022.1011049

**Published:** 2022-09-23

**Authors:** Nicola Luigi Bragazzi, Woldegebriel Assefa Woldegerima, Sarafa Adewale Iyaniwura, Qing Han, Xiaoying Wang, Aminath Shausan, Kingsley Badu, Patrick Okwen, Cheryl Prescod, Michelle Westin, Andrew Omame, Manlio Converti, Bruce Mellado, Jianhong Wu, Jude Dzevela Kong

**Affiliations:** ^1^Laboratory for Industrial and Applied Mathematics (LIAM), Department of Mathematics and Statistics, York University, Toronto, ON, Canada; ^2^Theoretical Biology and Biophysics Group, Los Alamos National Laboratory, Los Alamos, NM, United States; ^3^Department of Mathematics, Trent University, Peterborough, ON, Canada; ^4^School of Mathematics and Physics, University of Queensland, Saint Lucia, QLD, Australia; ^5^Vector-borne Infectious Disease Group, Theoretical and Applied Biology, Kwame Nkrumah University of Science and Technology, Kumasi, Ghana; ^6^Effective Basic Services (eBASE), Bamenda, Cameroon; ^7^Black Creek Community Health Centre, Toronto, ON, Canada; ^8^Department of Mathematics, Federal University of Technology, Owerri, Nigeria; ^9^Abdus Salam School of Mathematical Sciences, Government College University, Lahore, Pakistan; ^10^ASL Napoli 2 Nord, Naples, Italy; ^11^School of Physics and Institute for Collider Particle Physics, University of the Witwatersrand, Johannesburg, South Africa; ^12^Subatomic Physics, iThemba Laboratory for Accelerator Based Sciences, Somerset West, South Africa

**Keywords:** monkeypox, zoonotic disease, emerging and re-emerging infectious disease, underestimation, underreporting, under-detection, under-diagnosis, under-ascertainment

## Abstract

Monkeypox is an emerging zoonotic disease caused by the monkeypox virus, which is an infectious agent belonging to the *genus Orthopoxvirus*. Currently, commencing from the end of April 2022, an outbreak of monkeypox is ongoing, with more than 43,000 cases reported as of 23 August 2022, involving 99 countries and territories across all the six World Health Organization (WHO) regions. On 23 July 2022, the Director-General of the WHO declared monkeypox a global public health emergency of international concern (PHEIC), since the outbreak represents an extraordinary, unusual, and unexpected event that poses a significant risk for international spread, requiring an immediate, coordinated international response. However, the real magnitude of the burden of disease could be masked by failures in ascertainment and under-detection. As such, underestimation affects the efficiency and reliability of surveillance and notification systems and compromises the possibility of making informed and evidence-based policy decisions in terms of the adoption and implementation of *ad hoc* adequate preventive measures. In this review, synthesizing 53 papers, we summarize the determinants of the underestimation of sexually transmitted diseases, in general, and, in particular, monkeypox, in terms of all their various components and dimensions (under-ascertainment, underreporting, under-detection, under-diagnosis, misdiagnosis/misclassification, and under-notification).

## Introduction

Monkeypox is an emerging zoonotic disease caused by the monkeypox virus, which is an infectious agent belonging to the family of Poxviruses (*Poxviridae*), *Chordopoxvirinae* subfamily, and *Orthopoxvirus genus* (Hughes et al., 2010; Bunge et al., 2022). These viruses are large, brick-shaped, enveloped, double-stranded DNA viruses (Diven, 2001; Alakunle et al., 2020). Monkeypox virus is related to the *Variola* virus (VARV), the causative agent of smallpox, a life-threatening infectious disease fully eradicated in 1980, and another *Orthopoxvirus* (Barquet and Domingo, 1997; Riedel, 2005; Kmiec and Kirchhoff, 2022). Monkeypox has been endemic in some African countries, since 1970, when the first human case was reported in a 9-month-old child admitted to the Basankusu Hospital in the Democratic Republic of the Congo (DRC; Durski et al., 2018).

Currently, commencing from the end of April 2022, an outbreak of monkeypox is ongoing, with more than 43,000 cases reported as of 23 August 2022, involving 99 countries and territories across all the six World Health Organization (WHO) regions (Table 1). The most impacted WHO regions are the Region of the Americas (AMR; 52.0%) and the European Region (EUR; 47.5% of cases), followed by the Western Pacific Region (WPR; 0.3%), the African Region (AFR; 0.1%), the Eastern Mediterranean Region (EMR; 0.1%), and the South-East Asian Region (SEAR; 0.04%). On 23 July 2022, the Director-General of the WHO declared monkeypox a global public health emergency of international concern (PHEIC; Nuzzo et al., 2022), since the outbreak represents an extraordinary, unusual, and unexpected event that poses a significant risk for international spread, requiring an immediate, coordinated international response.

**Table 1:** Monkeypox cases (confirmed and suspected cases, deaths, and grand total) broken down according to the World Health Organization (WHO) region, and country.

**Country**	**Confirmed**	**Death**	**Suspected**	**Grand total**
**African Region (AFR)**	**54**	**1**	**7**	**62**
Benin	3	0	0	3
Ghana	46	1	0	47
South Africa	5	0	0	5
Uganda	0	0	6	6
Zambia	0	0	1	1
**Eastern Mediterranean Region (EMR)**	**35**	**0**	**8**	**43**
Iran	1	0	3	4
Lebanon	6	0	0	6
Morocco	1	0	0	1
Pakistan	0	0	1	1
Qatar	3	0	0	3
Saudi Arabia	6	0	0	6
Somalia	0	0	3	3
Sudan	2	0	1	3
United Arab Emirates	16	0	0	16
**European Region (EUR)**	**20,606**	**2**	**1**	**20,609**
Andorra	4	0	0	4
Austria	218	0	0	218
Belgium	624	0	0	624
Bosnia And Herzegovina	3	0	0	3
Bulgaria	4	0	0	4
Croatia	22	0	0	22
Cyprus	4	0	0	4
Czech Republic	39	0	0	39
Denmark	169	0	0	169
England	3,050	0	0	3,050
Estonia	9	0	0	9
Finland	22	0	0	22
France	2,873	0	0	2,873
Georgia	2	0	0	2
Germany	3,295	0	0	3,295
Gibraltar	6	0	0	6
Greece	50	0	0	50
Hungary	63	0	0	63
Iceland	12	0	0	12
Ireland	113	0	0	113
Israel	208	0	0	208
Italy	689	0	1	690
Latvia	4	0	0	4
Lithuania	5	0	0	5
Luxembourg	45	0	0	45
Malta	31	0	0	31
Moldova	2	0	0	2
Monaco	3	0	0	3
Montenegro	2	0	0	2
Netherlands	1,090	0	0	1,090
Northern Ireland	27	0	0	27
Norway	76	0	0	76
Poland	114	0	0	114
Portugal	810	0	0	810
Romania	34	0	0	34
Russia	1	0	0	1
Scotland	75	0	0	75
Serbia	31	0	0	31
Slovakia	12	0	0	12
Slovenia	43	0	0	43
Spain	6,117	2	0	6,119
Sweden	141	0	0	141
Switzerland	416	0	0	416
Turkey	5	0	0	5
Wales	43	0	0	43
**Region of the Americas (AMR)**	**22,531**	**4**	**28**	**22,563**
Argentina	72	0	0	72
Bahamas	2	0	0	2
Barbados	1	0	0	1
Bermuda	1	0	0	1
Bolivia	43	0	1	44
Brazil	3,895	1	7	3,903
Canada	1,168	0	11	1,179
Cayman Islands	0	0	1	1
Chile	207	0	2	209
Colombia	273	0	0	273
Costa Rica	3	0	2	5
Curaçao	1	0	0	1
Dominican Republic	6	0	0	6
Ecuador	19	1	1	21
Greenland	2	0	0	2
Guadeloupe	1	0	0	1
Guatemala	4	0	0	4
Haiti	0	0	1	1
Honduras	3	0	0	3
Jamaica	4	0	0	4
Martinique	2	0	0	2
Mexico	251	1	0	252
Panama	7	0	0	7
Peru	1,127	1	1	1,129
Puerto Rico	77	0	0	77
Saint Martin (French part)	1	0	0	1
United States	15,358	0	0	15,358
Uruguay	2	0	1	3
Venezuela	1	0	0	1
**South-East Asian Region (SEAR)**	**15**	**1**	**0**	**16**
India	9	1	0	10
Indonesia	1	0	0	1
Thailand	5	0	0	5
**Western Pacific Region (WPR)**	**122**	**0**	**0**	**122**
Australia	90	0	0	90
Japan	4	0	0	4
New Caledonia	1	0	0	1
New Zealand	4	0	0	4
Philippines	4	0	0	4
Singapore	15	0	0	15
South Korea	1	0	0	1
Taiwan	3	0	0	3
**Grand Total**	**43,363**	**8**	**44**	**43,415**

Data are extracted and collected from the Global Health Initiative (https://www.global.health/).

The epidemiological and clinical features of the ongoing monkeypox outbreak are different from those established for monkeypox since its initial isolation and identification and during the previous outbreaks, with sexual transmission suspected as the major transmission route and with the community of men having sex with men (MSM) disproportionately impacted (Liu et al., 2022; Thornhill et al., 2022). According to a large-scale study, out of 528 monkeypox infections diagnosed and reported from 16 countries, between April 27 and June 24, 2022, the transmission was hypothesized to have occurred more likely *via* sexual intercourse in 95% of the cases during the current outbreak (Thornhill et al., 2022). Other transmission routes include contact with infected animals and travel to endemic countries, occupational exposure, and social and household contacts (Liu et al., 2022). As such, monkeypox is not an exclusively sexually transmitted disease (STD), but its transmission has been hypothesized to be associated with sexual contact. This is an important distinction because we are still not sure that transmission is occurring through body fluids exchanged during sex, but rather it could be *via* contact with mucosal surfaces, scarification, or even respiratory exposures.

The real magnitude of the burden of disease could be masked by failures in ascertainment and under-detection. As such, underestimation affects the efficiency and reliability of surveillance and notification systems and compromises the possibility of making informed and evidence-based policy decisions in terms of the adoption and implementation of *ad hoc* adequate preventive measures. For example, another infectious outbreak, the still ongoing “Coronavirus Disease 2019” (COVID-19) pandemic was initially underestimated and this, along with the high degree of contagiosity of the virus, contributed to its quick global spread (Wu et al., 2020; Nakamoto and Zhang, 2021).

According to the working definitions of the “BCoDE-project” (Kretzschmar et al., 2012), underestimation can be due to various factors, including under-ascertainment. This can occur when infected subjects do not seek general practitioners or specialized health services, in that they perceive their illness as mild and/or self-limiting, do not have adequate health literacy and risk/disease perception, or they are asymptomatic and unaware of their disease status. Minority groups (including migrants, the lesbian/gay/bisexual/transgender/transsexual/queer/intersex, LGBTQI+, community, and other marginalized or difficult-to-reach communities) generally do not consult general practitioners or other healthcare workers (Kretzschmar et al., 2012). Cultural, religious, legal, administrative, economic, and financial factors can influence health-seeking behaviors. Underreporting, another component of underestimation, occurs when symptomatic cases in the community refer to health services but have their disease status not properly diagnosed or misclassified (under-diagnosis), or correctly diagnosed and classified but not effectively transmitted to the public health surveillance and monitoring bodies (under-notification). The reader is referred to Table 2 for further details.

**Table 2:** Underestimation, its components/dimensions with definitions and determinants.

**Failure to capture all cases**			**Definition**	**Determinants**
Underestimation	Under-ascertainment		Infected subjects do not seek health care	Health literacy, disease perception, perceived health needs, cultural and religious factors, legal, administrative, and financial barriers
	Underreporting	Under-diagnosis/under-detection	Disease status not diagnosed/misclassified	Measurement error, lack of knowledge concerning testing and/or interpretation of tests
		Under-notification	Diagnosis not transmitted to the surveillance and notification system	Reporting/notification policies

The topic of underestimation of monkeypox cases is of crucial importance in the field of public and global health. However, to the best of our knowledge, there exists no comprehensive review addressing the determinants underlying the underestimation of STDs, in general, and, in particular, monkeypox. Therefore, the present study was undertaken to fill this gap in knowledge.

## Materials and methods

An integrative review was conducted. Even though this technique dates back to the eighties, it is emerging as an innovative tool to synthesize and appraise the existing body of scholarly literature on the designated research problem/concept, enabling the combination of a heterogeneous array of sources, from empirical to conceptual/theoretical investigations, from quantitative to qualitative and mixed-method studies, and from observational to pilot, feasibility, and interventional studies (Broome, 2000). We employed this technique since we were able to identify and formulate a broad-scope research problem/concept/phenomenon of interest, particularly complex and articulated.

An integrative review enables to (i) overview and appraise theories and practices, (ii) to build bridges across diverse study fields, disciplines, and sectors, (iii) to generate and/or refine new knowledge and novel hypotheses, and (iv) to formulate and propose an actionable framework, being, as such, particularly suited for developing and informing healthcare policies and practices in an evidence-based fashion. More specifically, an integrative review study can be defined as “a review method that summarizes past empirical or theoretical literature to provide a more comprehensive understanding of a particular phenomenon or healthcare problem” (Broome, 2000).

To achieve the ambitious objectives of generating new knowledge and/or theories, an integrative review results in one or more of the following research synthesis forms: (i) a research agenda, (ii) a taxonomy or other conceptual classifications of constructs, (iii) alternative models or conceptual frameworks, and (iv) a metatheory/an array of metatheories.

Within the so-called “evidence synthesis ecosystem,” a systematic literature review and a meta-analysis have a highly focused, narrow research scope, whereas a scoping review has a broad research question and the objective of mapping, synthesizing, and combining the existing body of scholarly literature on the designated topic/research question.

**Figure 1:**
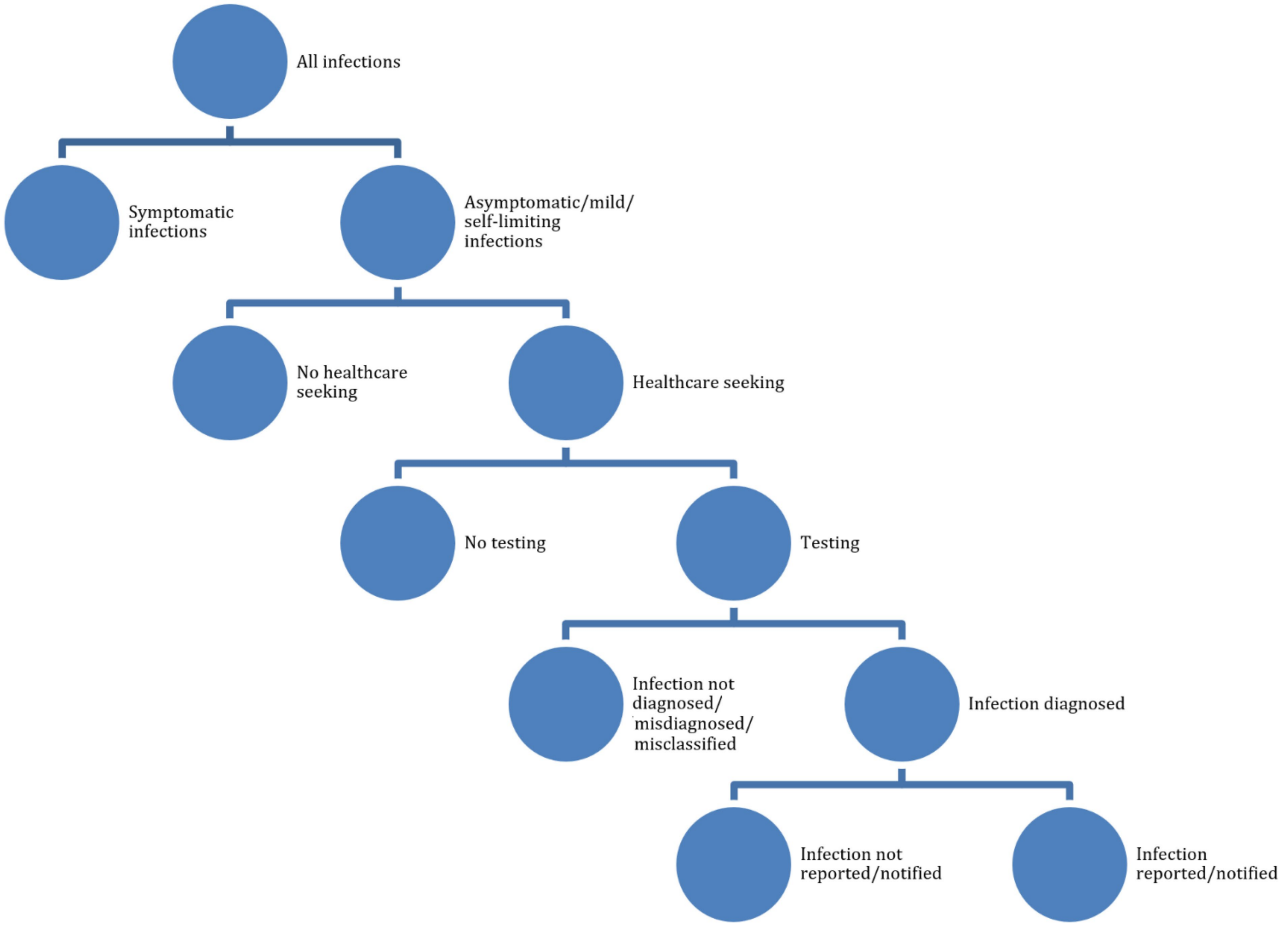
Pictorial flowchart of underestimation.

We searched a major scholarly electronic database, PubMed/MEDLINE, for papers without language filters, using a search string consisting of several components. First, these components were related to (i) health-seeking behaviors (awareness, knowledge, attitudes, practices, health-literacy, and health-seeking behavior), (ii) underestimation (under-ascertainment, underreporting, under-diagnosis, misdiagnosis/misclassification, under-detection, or under-notification), and, (iii) STDs (sexual transmission, sexually transmitted disease, or sexually transmitted infection). We wanted, indeed, to study determinants of underestimation of STDs, including healthcare-seeking behaviors. During a second round of literature search, we added a fourth component related to the LGBTQI+ community, since it is being particularly impacted by the current monkeypox outbreak (see Figure 1 and Tables 2, 3 for further details). Google Scholar was searched too, looking for resources and items not indexed yet at the time of the literature search and for ensuring a broader relevant coverage of the literature.

**Table 3:** Search strategy adopted in the present integrative review.

**Search strategy items**	**Details**
Keywords used in the search string	(“Health-seeking behavior” OR “health-literacy” OR “disease knowledge” OR “disease awareness” OR “disease perception” OR “risk perception”) (Underestimated OR underestimation OR underreporting OR underreported OR misreporting OR misreported OR under-diagnosis OR under-diagnosed OR under-ascertainment OR under-ascertained OR under-notification OR under-notified OR under-detection OR under-detected OR misclassification OR misclassified OR under-recognized OR under-recognition) (“Sexually transmitted infection” OR “sexually transmitted disease” OR “sexual transmission”) (LGBT OR LGBT+ OR LGBTQ OR LGBTQ+ OR LGBTQI OR LGBTQI+ OR “men having sex with men” OR “men who have sex with men” OR lesbian OR homosexual OR homosexuality OR bisexual OR bisexuality OR “sex and gender minorities” OR “sexual orientation” OR “gender identity”)
Time filter	From the onset for STDs and from the beginning of the monkeypox outbreak
Language filter	None applied

STD, sexually transmitted diseases.

## Results

### Underestimation of sexually transmitted diseases

Out of 230 items returned by searching PubMed/MEDLINE, 53 articles related to STDs (Franco, 1991; Koutsky et al., 1992; Lin et al., 1992; Schulte et al., 1992; Ashley et al., 1993; Webster et al., 1993; Brookmeyer et al., 1995; Maher and Hoffman, 1995; Petersen et al., 1995; Schachter and Chow, 1995; Wahdan, 1995; Schwebke et al., 1996; Agacfidan et al., 1997; Rompalo et al., 1997; Borisenko, 1998; Paget et al., 2002; Niccolai et al., 2005; Dhawan et al., 2006; Liu et al., 2006; Munson et al., 2008; Nguyen et al., 2008; Lusk et al., 2010; Andrea and Chapin, 2011; Hong et al., 2011; Roth et al., 2011; Wolfers et al., 2011; Koper et al., 2013; Krivochenitser et al., 2013; Oliffe et al., 2013; Gratzer et al., 2014; Mirzazadeh et al., 2014; Brown et al., 2015; Corbeto et al., 2015; Fakoya et al., 2015; Jenkins, 2015; Tomas et al., 2015; Johnson and Geffen, 2016; Kustec et al., 2016; Mlakar and Ramšak, 2016; Ni et al., 2016; Allard et al., 2017; Denison et al., 2017; Lee and Nishiura, 2017; Syme et al., 2017; Hall et al., 2018; Mangine et al., 2018; Shahesmaeili et al., 2018; Timsit et al., 2018; Steen et al., 2019; Knight et al., 2020; Moriña et al., 2021; Niekamp et al., 2021; Geba et al., 2022) were deemed eligible for inclusion in the present integrative review. More specifically, our comprehensive literature search enabled us to identify the following determinants of the underestimation of STDs: asymptomatic course (Wahdan, 1995; Shahesmaeili et al., 2018; Moriña et al., 2021); atypical clinical and epidemiological features (Ni et al., 2016), including atypical/unusual transmission routes (Allard et al., 2017; Lee and Nishiura, 2017; Timsit et al., 2018); differences in case definition (Schulte et al., 1992; Rompalo et al., 1997), and in regional/national testing rates (Koper et al., 2013; Kustec et al., 2016); underestimation among specific age groups, like the youth and the elderly, and populations, such as minority communities and visible racialized groups (Webster et al., 1993), migrant workers (Fakoya et al., 2015; Steen et al., 2019), sex workers (Agacfidan et al., 1997; Brown et al., 2015; Hong et al., 2011; Mirzazadeh et al., 2014; Shahesmaeili et al., 2018), or swingers (Niekamp et al., 2021); use of low-sensitivity and/or low-specificity diagnostic assays (Koutsky et al., 1992; Schulte et al., 1992; Ashley et al., 1993; Petersen et al., 1995; Schachter and Chow, 1995; Schwebke et al., 1996; Paget et al., 2002; Dhawan et al., 2006; Munson et al., 2008; Lusk et al., 2010; Andrea and Chapin, 2011; Gratzer et al., 2014), or inadequate clinical and microbe-isolation procedures (Koutsky et al., 1992; Lin et al., 1992); inadequate STD screening policies/protocols (Wahdan, 1995; Lusk et al., 2010; Roth et al., 2011; Corbeto et al., 2015; Geba et al., 2022); measurement error/misclassification (Franco, 1991; Krivochenitser et al., 2013; Tomas et al., 2015); barriers to accessing STD testing and management services (Mlakar and Ramšak, 2016; Denison et al., 2017), including psychological issues (Oliffe et al., 2013), or lack of available facilities and infrastructures in resource-limited contexts (Maher and Hoffman, 1995); self-treatment (Borisenko, 1998); disease perception/health literacy (Liu et al., 2006; Nguyen et al., 2008; Wolfers et al., 2011; Hall et al., 2018), including risk perception (Syme et al., 2017), that is to say, the subjective assessment about the characteristics and severity of a given risk; and limited/strained testing and diagnostic capacity (Schulte et al., 1992).

These studies concerned the following sexually transmitted pathogens/STDs: herpetic diseases (Koutsky et al., 1992; Ashley et al., 1993), human papillomavirus or HPV (Franco, 1991; Brown et al., 2015; Shahesmaeili et al., 2018; Moriña et al., 2021), chancroid (Schulte et al., 1992), *Chlamydia trachomatis* (Lin et al., 1992; Maher and Hoffman, 1995; Schachter and Chow, 1995; Agacfidan et al., 1997; Paget et al., 2002; Krivochenitser et al., 2013; Corbeto et al., 2015; Tomas et al., 2015; Kustec et al., 2016; Mlakar and Ramšak, 2016), syphilis (Webster et al., 1993; Gratzer et al., 2014; Shahesmaeili et al., 2018) and genital ulcer disease (GUD; Rompalo et al., 1997), gonorrhea (Webster et al., 1993; Maher and Hoffman, 1995; Borisenko, 1998; Krivochenitser et al., 2013; Tomas et al., 2015; Shahesmaeili et al., 2018), trichomoniasis (Maher and Hoffman, 1995; Petersen et al., 1995; Munson et al., 2008; Lusk et al., 2010; Andrea and Chapin, 2011; Roth et al., 2011; Tomas et al., 2015; Shahesmaeili et al., 2018), bacterial vaginosis (Schwebke et al., 1996), *Ureaplasma urealyticum* (Dhawan et al., 2006), Zika virus (Allard et al., 2017; Lee and Nishiura, 2017), amebiasis (Timsit et al., 2018), and human immunodeficiency virus, or HIV (Wahdan, 1995; Liu et al., 2006; Nguyen et al., 2008; Mirzazadeh et al., 2014; Fakoya et al., 2015; Ni et al., 2016; Hall et al., 2018; Steen et al., 2019).

Three articles (Niccolai et al., 2005; Jenkins, 2015; Mangine et al., 2018) contained recommendations to overcome these shortcomings: namely, (i) to use sensitive and specific assays, (ii) to accurately collect sexual history, including data related to sexual orientation, and identify high-risk sexual behaviors (Jenkins, 2015), (iii) to strengthen sentinel surveillance and establish further sites, to improve the quality of collected data, (iv) to deploy and link multiple data sources, such as self-reports, medical record reviews, and regional/state health department reports, harmonizing, when appropriate, the various and different reporting systems and case definitions (Niccolai et al., 2005), and, (v) to exploit the web, including social media and social networks to recruit high-risk populations, like the MSM community (Mangine et al., 2018).

Three other studies (Brookmeyer et al., 1995;Johnson and Geffen, 2016; Knight et al., 2020) focused on mathematical modeling, suggesting that the underestimation of STDs can occur when one fails to properly model high-risk sexual behaviors (such as unprotected, condomless sexual intercourse, use of recreational drugs or chemsex, sex with commercial partners, or with individuals the HIV status is unknown; Johnson and Geffen, 2016; Knight et al., 2020) or does not adjust for the follow-up bias (potential losses during the follow-up; Brookmeyer et al., 1995).

Specifically concerning behavioral determinants of STDs (i.e., healthcare-seeking behaviors), a series of qualitative in-depth interviews carried out among 24 university students, exhibiting risky sexual behaviors (Denison et al., 2017), identified three main types of barriers to STD testing: (i) personal (underestimation of risk, perception of STD as a not serious disease, fear of invasive procedures, self-consciousness in genital examination, and/or being too busy); (ii) structural (economic-financial cost of testing, environment–clinician attributes and attitudes); and, (iii) social (concern/fear of stigmatization).

Finally, seven of the 53 retrieved articles focused on the MSM community (Liu et al., 2006; Koper et al., 2013; Brown et al., 2015; Mlakar and Ramšak, 2016; Hall et al., 2018; Mangine et al., 2018; Knight et al., 2020).

### Underestimation of monkeypox cases

So far, the only attempt to test the hypothesis of the impact of stigmatization on monkeypox case reporting in European countries has been done by Kenyon (Kenyon, 2022), employing Spearman’s correlation test to quantitatively explore whether the monkeypox national cumulative incidence was negatively associated with the intensity of screening for STIs and a composite indicator of LGBTQI+ rights (the “Rainbow Index”). The author found, instead, a positive correlation between the monkeypox epidemiological trend and the intensity of chlamydia/gonorrhea (rho 0.68, *p* < 0.0001), and syphilis (rho 0.62, *p* < 0.0001) screening, and the Rainbow Index (rho 0.65, *p* < 0.0001), suggesting that in several Eastern European countries, the real burden of monkeypox is underestimated.

Besides stigmatization and related issues, a few monkeypox infections are asymptomatic (Fleischauer et al., 2005; Karem et al., 2007; Guagliardo et al., 2020) and, when present, symptoms are atypical, in that this outbreak differs from previous outbreaks, in terms of a shift in mean age and the most affected age group, affected sex/gender, risk factors, clinical course, signs/symptoms, and, above all, the sexual transmission route (Bragazzi et al., 2022a). As such, physicians may not recognize the infection as monkeypox. A recent “knowledge, attitudes, and practices” (KAP) survey among Italian physicians showed unsatisfying monkeypox-related knowledge and attitude levels (Riccò et al., 2022). For example, systemic complications of monkeypox, especially among children, were generally largely overlooked. Of note, Italian physicians who took part in the survey showed substantial uncertainties and knowledge gaps related to monkeypox, in terms of clinical presentation and main features, risk factors, and preventative measures, with less than one-fifth of them confident in properly recognizing incident monkeypox cases during their clinical activities. Another survey conducted in Jordan (Sallam et al., 2022), among 615 university students in health schools/faculties (medicine, nursing, dentistry, pharmacy, medical laboratory sciences, and rehabilitation), identified serious gaps in knowledge, with only three out of 11 monkeypox-related knowledge items identified correctly by >70% of the respondents. Only 26.2% of the participants knew that monkeypox is a vaccine-preventable disease. However, information about knowledge of monkeypox among physicians and allied health professionals is scarce.

Also, the monkeypox case definition has only recently been revised to be adapted to the ongoing outbreak, in order to reflect the new findings and clinical and laboratory features (Bragazzi et al., 2022a; Centers for Disease Control and Prevention (CDC), 2022).

Another factor that could result in monkeypox underestimation is testing and diagnostic capacity, with a general lack of point-of-care tests currently available and, in some countries, overall testing (Nuzzo et al., 2022). Diagnostic/testing capacity for monkeypox varies substantially worldwide—some countries like the United States are able to process up to several thousand specimens *per* week (Cohen, 2022), while others have no diagnostic capacity at all; moreover, testing and diagnostic capacity are further strained by the still ongoing COVID-19 pandemic. Testing includes non-*variola Orthopoxvirus* (NVO) generic real-time polymerase chain reaction (PCR) test, monkeypox-specific PCR, and sequencing (Jiang et al., 2022).

Further, services and healthcare provisions offered by sexual health clinics in some countries, like the United Kingdom, are being significantly impacted and disrupted. This could result in a significant delay in the diagnosis, treatment, and reporting of cases.

Finally, in most cases, contact tracing (also known as partner notification) is unfeasible or presents particular challenges in the MSM community, given that contacts of infected individuals are casual sexual partners (Bell and Potterat, 2011; Bragazzi et al., 2022a).

## Discussion

Sexually transmitted diseases are generally overlooked and underestimated (Sartorius, 2007; Bragazzi et al., 2022b). Based on our integrative review of the literature, monkeypox case underestimation could be significant. This has important implications for public and global health providers as well as policy- and decision-makers, epidemiologists, and mathematical modelers.

According to Andersen’s “Behavioral Model of Health Services Use,” health-seeking behaviors are complex and multidimensional, depending on an array of factors, including “predisposing factors” (such as age, sex/gender, ethnicity, or cultural and social variables), “enabling factors” (like financial variables—insurance coverage—or healthcare accessibility/availability), and “need factors” (health, risk, and disease perceptions, health literacy, medical conditions, or underlying co-morbidities; Babitsch et al., 2012). Symptoms of some STDs can be mild and individuals may not seek healthcare. Moreover, in the LGBTQI+ community, STDs are usually perceived as a “part of the way of life” and as inconvenient consequences of being sexually active. In the pre-HIV pre-exposure prophylaxis (PrEP) era, HIV was considered the most anxiety-provoking STD, followed by viral, recurring STDs, and bacterial STDs, which were conceived as trivial and treatable. On the other hand, while not generating particular concerns in terms of disease perception, a diagnosis of STDs was associated with feelings of being “dirty and ashamed” (Holt et al., 2010). Risk and disease perceptions regarding HIV have changed after PrEP introduction, but the general thought that STD is an untoward consequence of sexual activities has remained practically unchallenged. Intended and actual utilization of healthcare provisions has been found to be related to the endorsement of stigmatization of certain sexual practices, such as anal sexual intercourse (Kutner et al., 2022). Awareness and attitudes toward STDs are highly heterogenous among MSM, with some infections considered scarier and others less, depending on their transmission mechanisms, epidemiology (prevalence), visibility of symptoms, and impact on health, as well as the availability of vaccines and treatment options, based on both personal or friends’ experiences (Datta et al., 2019).

Sexual health clinics are usually the first point of access in the case of STDs. However, some sex and gender minorities (SGMs), despite being at higher risk for STDs, including monkeypox, could be underrepresented. Holmes and Beach (2020) found that individuals self-identifying as bisexuals were approximately one-quarter of sexual health clinic users, while they represent more than half of SGMs populations. The so-called “bisexual erasure” or “bisexual invisibility” may be one of the factors explaining the potential underestimation of monkeypox cases, with the number of cases reported among men having sex with men and women (MSMW) being tremendously underestimated.

All these behaviors can be explained utilizing the “minority stress theory” (MST), according to which some marginalized communities subjected to stigmatization and discrimination experience more stressors than the general population, resulting in increased stress-linked coping behaviors, substance use, encounters with random/casual sex partners, and poorer health outcomes and health-related inequalities. Health disparities could be due to lower access to healthcare services, including preventative and STD screening/testing ones (Holmes and Beach, 2020).

There are different interests and actors at stake and a holistic approach is required to address STDs, in general, and monkeypox, specifically. To really advance the field of STD- and sexual health-related research, institutional and governmental bodies should facilitate “sex-at-birth, sexual orientation, and gender identity” (SSOGI)-related data collection, dissemination, and utilization, to favor a more “inclusive STD reporting” (Baptiste-Roberts et al., 2017). Currently, SSOGI data collection is not routinely implemented, with the risk of invisibilizing individuals with bi/bi+ umbrella labels, such as bisexual, queer, and pansexual individuals (Baptiste-Roberts et al., 2017). Several LGBTQI+ organizations have been collecting SSOGI data, but current public health surveillance systems are not updated to incorporate such information (Baptiste-Roberts et al., 2017). Of note, a major shortcoming of the investigation by Kenyon (Bragazzi et al., 2022b) is that the incidence of monkeypox cases was computed utilizing the entire (general) population, rather than the MSM/SGM/LGBTQI+ population. The latter point reflects the challenges that can be encountered in measuring and collecting data related to the sexual orientation/gender identity of a patient, given that there exist several socio-cultural, historical, as well as political implications underlying these issues. Data collected by healthcare providers are affected by the patient’s willingness to disclose personal, sensitive information and their degree of openness, while self-report data suffer from selection/self-selection biases. As such, the real size of the MSM/SGM/LGBTQI+ population remains unknown and discrepancies among studies and differences among countries point to the influence of societal variables as well as the precise definition of what the MSM/SGM/LGBTQI+ population is (Marcus et al., 2013).

Specifically, concerning monkeypox cases, even though in a few cases, systemic prodromal symptoms (like fever, headache, lymphadenopathy, etc.) typical of the invasion period may be missing, with visible symptoms appearing during the skin eruption stage and a few asymptomatic individuals described in the current as in previous outbreaks, there are good reasons to suspect underestimation just by looking at data, since, as noted by Nuzzo et al. (2022), the United States, despite having a larger population size, have reported fewer cases than the United Kingdom.

The engagement of the LGBTQI+ community, and especially of bisexual/pansexual (bi/bi+) populations, with community-based sexual health providers is of paramount importance (Baptiste-Roberts et al., 2017) to offer LGBTQI+-tailored sexual health services. Scaling up community outreach and recruitment of LGBTQI+ members, including bi/bi+ people to engage in sexual health services represent challenges that need to be prioritized (Baptiste-Roberts et al., 2017). Adopting an intersectional lens, with a focus on populations reporting multiple stigmatization and discrimination, such as non-White communities, is crucial to address unmet needs. Educating staff to be culturally sensitive and competent, fighting against systemic and institutional stigmatization, and homo-bi-trans-phobia, and creating an inclusive environment represent another societal onus. Institutional bodies should conduct awareness campaigns to enhance health literacy, minimize structural or perceived barriers to STD testing, develop effective and innovative strategies aimed at addressing personal beliefs and improving STD testing rates, and favor the adoption of healthy sexual practices and behaviors (Pitts, 2020).

Social media, including news outlets, should also play their role in changing societal views of STDs (Pitts, 2020), combating disinformation and infodemic (Ennab et al., 2022), and creating awareness that monkeypox can infect all humans regardless of their age, sex/gender, sexual orientation, or gender identity. Moreover, there are various factors that may increase the potential risk for exposure, including close, sexual, and/or intimate contact with someone who has monkeypox and symptoms, such as rash, soreness, or scabs. Potentially, any sexually active individual could contract the infection, even if the focus is mainly on the MSM community. This could lead to a (further) underestimation of infectious transmission among other populations, as previously mentioned.

## Conclusion and future prospects

Monkeypox is an emerging sexually transmitted infection, which is representing a global public health concern. Mathematical modeling of monkeypox should adjust for the underestimation of cases and public and global health policy- and decision-makers should consider the “hidden burden” of monkeypox when designing and implementing packages of interventions. Studies are urgently needed to quantify the degree of underestimation of monkeypox cases to better inform the responses to the outbreak.

## Author contributions

JW and JDK conceived and drafted the paper. WAW, SAI, QH, XW, AS, KB, PO, CP, MW, AO, MC, and BM critically revised it. All authors contributed to the article and approved the submitted version.

## Funding

This research is funded by Canada’s International Development Research Centre (IDRC) and the Swedish International Development Cooperation Agency (SIDA; Grant No. 109559–001). NLB and JDK acknowledge support from IDRC (Grant No. 109981), New Frontier in Research Fund-Exploratory (Grant No. NFRFE-2021-00879), and NSERC Discovery Grant (Grant No. RGPIN-2022-04559). AS would like to thank The University of Queensland’s AI4PAN research group. Portions of this work were performed at the Los Alamos National Laboratory under the auspices of the US Department of Energy contract 89233218CNA000001 and supported by NIH grant R01- OD011095 (SAI).

## Conflict of interest

The authors declare that the research was conducted in the absence of any commercial or financial relationships that could be construed as a potential conflict of interest.

## Publisher’s note

All claims expressed in this article are solely those of the authors and do not necessarily represent those of their affiliated organizations, or those of the publisher, the editors and the reviewers. Any product that may be evaluated in this article, or claim that may be made by its manufacturer, is not guaranteed or endorsed by the publisher.
